# Mistrust in marriage-Reasons why men do not accept couple HIV testing during antenatal care- a qualitative study in eastern Uganda

**DOI:** 10.1186/1471-2458-10-769

**Published:** 2010-12-17

**Authors:** Elin C Larsson, Anna Thorson, Xavier Nsabagasani, Sarah Namusoko, Rebecca Popenoe, Anna Mia Ekström

**Affiliations:** 1 Division of Global Health/IHCAR, Department of Public Health Sciences, Karolinska Institutet, Stockholm, Sweden; 2 Makerere University School of Public Health, Kampala, Uganda

## Abstract

**Background:**

A policy for couple HIV counseling and testing was introduced in 2006 in Uganda, urging pregnant women and their spouses to be HIV tested together during antenatal care (ANC). The policy aims to identify HIV-infected pregnant women to prevent mother-to-child transmission of HIV through prophylactic antiretroviral treatment, to provide counseling, and to link HIV-infected persons to care. However, the uptake of couple testing remains low. This study explores men's views on, and experiences of couple HIV testing during ANC.

**Methods:**

The study was conducted at two time points, in 2008 and 2009, in the rural Iganga and Mayuge districts of eastern Uganda. We carried out nine focus group discussions, about 10 participants in each, and in-depth interviews with 13 men, all of whom were fathers. Data were collected in the local language, Lusoga, audio-recorded and thereafter translated and transcribed into English and analyzed using content analysis.

**Results:**

Men were fully aware of the availability of couple HIV testing, but cited several barriers to their use of these services. The men perceived their marriages as unstable and distrustful, making the idea of couple testing unappealing because of the conflicts it could give rise to. Further, they did not understand why they should be tested if they did not have symptoms. Finally, the perceived stigmatizing nature of HIV care and rude attitudes among health workers at the health facilities led them to view the health facilities providing ANC as unwelcoming. The men in our study had several suggestions for how to improve the current policy: peer sensitization of men, make health facilities less stigmatizing and more male-friendly, train health workers to meet men's needs, and hold discussions between health workers and community members.

**Conclusions:**

In summary, pursuing couple HIV testing as a main avenue for making men more willing to test and support PMTCT for their wives, does not seem to work in its current form in this region. HIV services must be better adapted to local gender systems taking into account that incentives, health-seeking behavior and health system barriers differ between men and women.

## Background

In 2002 the World Health Organization (WHO) formulated recommendations advising couple HIV testing in settings with high HIV prevalence [1,2]. The policy guidelines are based on the assumption that couple testing would help increase spousal support for women to use prevention of mother to child transmission (PMTCT) services, create opportunities for secondary prevention by counseling both men and women about HIV, and increase the uptake of testing and identification HIV infected persons [3-5].

In 2006, following the WHO policy, Uganda's Ministry of Health formulated PMTCT guidelines that promoted couple HIV testing [4,6]. Mother to child transmission (MTCT) accounted for 16% of new HIV infections in Uganda in 2008, despite a reported coverage of PMTCT services of 50%[7]. PMTCT is a short-term and relatively inexpensive intervention that can reduce MTCT rates to just over 1%, and therefore it is vital to find effective ways to increase women's access to and completion of PMTCT services [7].

However, the uptake of couple HIV testing during ANC in SSA, including Uganda, has so far been very low, only 5-12% of pregnant women are HIV tested with their spouses [8-12]. Studies from SSA have shown an association between male partner involvement in their wives' ANC, couple HIV testing, and, pregnant women's likelihood of accessing and completing PMTCT services [9,10,13,14]. These associations led to the assumption that couple testing would be key for increasing male partners' involvement in PMTCT. Instead, it seems more likely that preexisting male partner involvement is what leads some men to accompany their wives to ANC and to support them during PMTCT, and it is possible that the few men who accompany their wives to ANC are not representative of most men.

Men's uptake of HIV testing is lower than women's in most low and middle-income countries since women are tested more often when pregnant, and as a consequence men also have less access to antiretroviral treatment [5,6,15,16]. In Uganda, virtually all adult men (99%) have heard of HIV, but only 11% have ever tested for HIV, while 57% of all pregnant women have been HIV tested [17-22]. The low testing rate among men in Uganda is disappointing since HIV was first acknowledged by the Ugandan government already in the late 1980's, and large-scale information campaigns about HIV and prevention, including HIV testing, have been carried out at all levels of society [17,23].

A recent study of pregnant women's experiences of the implementation of the couple HIV testing policy in the same population, revealed that many women felt subordinate to their partners and burdened by health workers' demand to bring their male partners in for HIV testing[24].

Couple HIV testing during ANC has obvious potential benefits. However, the poor uptake of couple testing and the difficulties of reaching men suggest that the current approach needs to be reevaluated. The present study examines men's own perspectives by exploring fathers' views on, and experiences of couple HIV testing in two rural Ugandan districts.

## Methods

### Study setting

The study was conducted in Iganga and Mayuge Districts, Busoga region, eastern Uganda. More than 80% of the population in the two districts lives in rural areas and only Iganga town is semi-urban. The majority of the population are subsistence farmers, but in Iganga town many are also involved in small businesses such as selling vegetables and fruits in the market (mostly women) or working as bicycle taxi drivers (only men). The literacy rate among men in the region is in line with Uganda's national rate of nearly 90%. Approximately half of the population is Muslim and half is Christian. Polygamy is common irrespective of religion. In most families, men are the breadwinners and the decision-makers [25]. Although "zero-grazing" and "Western" norms of monogamy have been communicated through the media, and are seemingly becoming the norm, many men still adhere to the traditional practice of having several concurrent partners [23,25].

The total fertility rate is high at 6.7 and is similar to the Ugandan average. About 70% of the population lives within of 5 km from a health unit. The HIV prevalence in Busoga region is about 6.2% among women and 4.4% among men [17]. Free HIV testing, including couple HIV testing during ANC should be offered at hospitals and most health centers in the districts. The lowest level health facilities without onsite HIV testing are supposed to refer couples for testing [6]. A number of non-governmental organizations occasionally provide HIV testing through outreach activities in the communities. According to national guidelines, counseling that addresses people's concerns and fears should precede all HIV testing.

### Study participants and data collection

Study participants were fathers from both rural and semi-urban locations who had had a child in the past year. Data were collected through nine focus groups with about 10 participants each, four held in April 2008 and five held in April 2009, as well as through 13 in-depth interviews held in April 2009, until saturation was achieved. An iterative process was used whereby the data from 2008 were reviewed and preliminary analysis carried out before the next data collection. This preliminary analysis enabled the team to explore emerging themes and issues more in detail.

Participants in the 2008 FGDs were homogenous with regard to place of living (two in rural and two in semi-urban settings) and then sub-divided based on number of children (one child vs. two or more children) in order to capture views from men with similar potential ANC exposure. Both in 2008 and 2009, all FGDs included both men who had and who had not been HIV tested. FGDs were held at times and venues considered convenient by the participants, usually under a big tree in the middle of the village. Study participants were in each data collection location recruited by a Ugandan research assistant together with a local leader (local council chairperson). The two together found eligible men at peoples' homes and at places where people normally gather. Recruitment took place at different times of the day to get both those who worked outside the home and farmers working at home. The study specifics were explained to eligible men and they were invited to participate.

For the FGDs in 2009 we used the same recruitment procedures and similar inclusion criteria as in 2008 except for the criteria of the number of children, where we instead used years in stable relationship with partner (less than vs. more than five years). The in-depth interviews were conducted after the FGDs in order to explore personal experiences around HIV and HIV-testing and to follow up on issues identified in the FGDs. As with the recruitment of FGD participants the aim was to include both rural and semi-urban men who had had a child within the last year. We also sought men with a range of experiences of HIV-testing: HIV tested during ANC, HIV tested but not during ANC, and never HIV tested. Interviews were carried out at times and places that ensured privacy and that were convenient for the respondents, typically at their home or work place.

The data collection instruments were topic guides modified according to each data collection method, as well as to the type of respondents in the interviews. The topic guides comprised key areas such as views and experiences of couple HIV testing during ANC and HIV testing in general, experiences with and perceptions of ANC attendance, and factors hindering or facilitating couple testing and ANC attendance. One male and one female sociologist collected data in the local language, Lusoga. FGDs and interviews were audio-recorded, and thereafter translated and transcribed in English. A note-taker participated in the FGDs to capture the group dynamics, gestures, and emotions of participants.

The atmosphere during the FGDs was open and men seemed to enjoy discussing this topic. They were interested in knowing more and the research team often stayed after the discussion to answer questions about various issues related to HIV. Men commonly asked questions about how they could obtain condoms, how one use them, if they could use the same condom only once, and why they should be HIV tested before developing symptoms.

### Analysis

Data were analyzed using latent content analysis as described by Graneheim and Lundman [26]. All through the data collection periods the author ECL was present in the field. For the first phase of data collection she joined the discussion before and after the formal FGDs, and during the second phase she and author XN observed all FGDs. FGDs were discussed in detail by the research team immediately after each session to guide subsequent FGDs, and to identify issues that should be followed up in the interviews. This initial discussion also helped establish when saturation had been reached. After data collection, ECL read all transcripts repeatedly, and written comments were made on the texts. All authors then read selected transcripts. Within the texts meaning units (i.e. individual sentences related to a certain concept) were identified. Meaning units were then condensed and assigned codes. Initial coding was done manually on paper copies of transcripts, and thereafter handled and organized using Nvivo software 8.0. The codes were compared and grouped into categories with similar topics, which were in turn grouped into themes. The themes developed were developed based on the interpretation of underlying meaning on a higher analytical level as compared to the more descriptive categories. All steps of the analysis were discussed among the co-authors.

Triangulation in the analysis of the data was achieved by having researchers with many different backgrounds analyze the data separately (pharmacist, epidemiologist, M.D., anthropologist, sociologist). Another source of validation was through presenting the results on a feedback workshop to people living in the study area.

### Ethics

Ethical approval was sought and obtained from Makerere University School of Public Health Institutional Review Board and the Uganda National Council of Science and Technology. Eligible men were informed that their participation was entirely voluntary and all signed an informed consent form when they agreed to participate in the study.

## Results

We identified a number of reasons for the low uptake of couple HIV testing, including lack of understanding of why testing was important, the perceived stigmatizing nature of HIV care, and perceived rude attitudes among health workers. Most prominent, however, was men's descriptions of their marriages as fundamentally unstable and distrustful, making the idea of couple testing unappealing because of the conflicts it could lead to in marriages. Secondary prevention of HIV or partner support was not stated reasons to test. The men's answers did not differ by place of residence or age.

### Mistrust in marriages hinders men from accepting couple HIV testing

During the discussions and interviews, the men often talked about the nature of their marriages. They rarely portrayed marriages built on love and understanding, instead they described relationships where mistrust was widespread, and extramarital affairs were common, especially among men. Extramarital affairs were in general tacitly accepted within a marriage, and rarely discussed between spouses. However, the ever-present suspicion, that one's partner would be unfaithful, created a pervasive atmosphere of mistrust between husbands and wives. It was nevertheless evident that the men valued family harmony and were unwilling to do anything that could provoke outright family conflict.

The men had a generally negative view of couple HIV testing and the "promise" of couple HIV testing at ANC health facilities actually discouraged men from accompanying their wives on antenatal visits. In fact, the men saw the situation where one might receive HIV-test results in the presence of one's spouse as a disadvantage rather than as an advantage:

*You know in most cases, we men don't like to accompany our wives because we know about this program whereby once you go with your wife, then you must be tested for HIV. So we are very worried about escorting our wives (for ANC)*.

In-depth interview participant, henceforth "IDI"

*We are told that when you go for ANC, they screen you for HIV, syphilis and other diseases. So this makes one fear*.

Focus group discussant, henceforth "FGD"

Instead men felt that voluntary individual testing was preferable, because it would enable them to keep a positive test result from their wives, if they felt it necessary.

We do not want to disclose our status to our partners; when you go for HIV testing you even never tell your wife that you went. So you and your wife test in different places. (FGD)

Since trust and love were uncommon ingredients in a marriage, disclosure that one or the other spouse was HIV positive would not elicit supportiveness in the other spouse, men explained, but rather would risk creating a lot of arguing and could easily disturb a fragile family harmony.

It is because we don't tell our wives the truth; you lie to her that she is the only one yet her friends see you with other women. So if we go for testing and find out that we are infected she will immediately start quarrelling since already there is no love that we show each other. (FGD)

The ever-present possibility that either member of the couple was having an extramarital affair only made the "offer" of couple HIV testing and the risk of discordance more unappealing, as the following men explained:

This issue is big, the root cause of all these problems is the issue of adultery, couples are not open to each other and therefore fear to discuss those things together/.../(FGD)

*It affects us negatively; for example if you have been together for 10 years and they tell you that she is infected and you are not, it causes misunderstanding in the home/.../*.

It brings a lot of thoughts in a home, you really get strong thoughts...you start wondering where this person got the infection. You really live in total/.../You wonder a lot where she got this infection and you may even develop diseases like high blood pressure. (FGD)

Since men were, by general agreement, the ones who were more likely to be having sexual relationships outside of the marriage, the risk of testing, getting a positive result, and having it made known to their spouses was perceived as especially threatening.

It is because men have so many extramarital relationships. They lie to their wives that they are faithful, but actually they have many women, not wives but women with whom they have sexual relationships. So when they think about the women they have had intercourse with, they choose to rather stay in the dark without finding out their HIV status. (FGD)

*The wife feels so bad because in everyday life women know that it is we men who are promiscuous, so she would straight away know that I got infected from the places I visit, and from that moment my wife never stays. She would say that I am going to infect her with HIV*.

Men's portrayed tendency to deceive their wives was supported by the power structure of marriages in which men are decision-makers and have the power over their wives' actions. Men resisted women's efforts to influence them, including when it came to HIV-testing:

We men have a despising heart; a woman will tell you to go to the health center with her, but when you get up in the morning you tell her not to disturb you claiming that you are going to look for money. In that way you avoid her. (FGD)

As to why some men did go for couple HIV testing, the men we interviewed speculated that these were men who had a good relationship with their wives, and had marriages marked by mutual love, trust, and understanding.

Few men go for testing, and only those who are close to one another get tested (together with their wife), because they encourage each other/.../

It depends on the way you believe/live in the home, a man may say that if you are willing to go, you go, but as for me I won't. But it should be that there is a good relationship between wife and husband, that's when she tells you to go and you go. (FGD)

In other cases the men emphasized a sense of responsibility to their wives and families, along with love:

To me what forces me is my responsibility. I fear to spoil my love, a problem to come and it is a result of my failure to escort my wife, so it gives me the responsibility to go with her when she starts to go for antenatal care, and they tell us, and I do what they have told us. And also because of the love, I have with her. (FGD)

### Men can recite HIV testing messages but this does not induce them to test

When speaking in the abstract about HIV testing, men recognized its importance. However, despite the ability to recite the HIV testing messages broadcasted in their communities, they did not see any reason to get tested themselves. Most of the men in the study had not been tested and only very few had been tested during ANC. Among the men who had never tested, some went so far as to agree that it would be good to get tested, but most reasoned firmly that they did not want to get tested at all.

How was it that they could recite the messages about HIV testing but not see any reason why they themselves should actually go for testing? A topic that came up in all FGDs and interviews was that men wanted more convincing arguments for why they should be tested. The men described again and again how they had been informed that they should be tested but not why it would be advantageous to identify a possible HIV infection before symptoms arise.

I could say that people need to be informed thoroughly, rather than telling them, friends "come for blood testing, come for blood testing". But we have to sit down and teach people that a person should have his/her blood tested when he/she is still healthy (meaning without symptoms). (IDI)

That men lacked the kind of information they needed to motivate HIV testing was further reinforced by the men's questions and curiosity around HIV-related issues in the discussions that immediately followed the interviews and FGDs.

The media, especially the radio, which was the men's main source of information about HIV, had conveyed the message that one should get tested, but the campaigns had apparently failed to motivate the men to do so. The men suggested that live information campaigns at the community level instead of frequent media messages would be a more effective strategy for getting them to test, in part because it would enable them to ask questions.

These programs are mostly on radio, but it would be better if you organized meetings on the LC level (i.e. in the community), rather than putting programs on the radio/.../then I would come there and I will understand everything better. (IDI)

Men also suggested that men who had been HIV tested should serve as role models, talking about their experience to other men. This strategy, men felt, would have a greater impact on their willingness to get tested than radio messages. Along the same lines, they also suggested they might be more motivated to test if men who where HIV positive came and talked to them about their lives.

Like in our village, there is no person who has ever been ill to the extent of dying as a result of HIV/AIDS. So it is needful for you to get somebody who had suffered a lot from HIV/AIDS and you walk with him as a testimony for the people, to let them understand that this (HIV) cannot kill a person if he gets good medicine and care. (FGD)

Interestingly, none of the respondents mentioned secondary prevention, neither to the wife nor to extramarital partners, as a motive for HIV testing. This is in line with their description of their marriages where concern for their wives' well being did not seem to dissuade many men from having unprotected sex outside the marriage.

The expressed need for more information and different kinds of information to motivate testing in the community was reinforced by the story one focus group participant told of finally being convinced to get tested:

I had never been tested, but we were taught and we finally went for testing after 2 days. So the issue is having people taught first so as to be strong in heart in that they give you time with some examples given to you and after which you are tested, that even if you are found infected, you have a look at your sick colleague and get encouraged because I took some given days being taught and as a result I become strong and went for testing. (FGD)

### Perceived deficiencies and problems in the health care system discouraged the men from testing

The problems men raised with the health care facilities were many, but can be divided into four overarching categories: the distance and cost of getting there, organizational problems at the facilities, the perceived rudeness of many health care workers, and the perception that informal payment was required to obtain medicine and good care.

Health facilities were often far away from where the men lived, and going there was not only costly, but took time away from work and other activities:

Now, this becomes a long distance/.../in order for the person to go and have a blood test/.../it is just a long distance. (IDI)

Furthermore, when one finally got to the health facility, organizational problems made it cumbersome to actually get the care one needed. Men reported having been forced to wait an entire day for care, a heavy sacrifice for someone who needs to work to support his family:

Even the long line at the hospital (chorus answer yes....) women can be there from 08.00 a.m. in the morning up to evening, and waste all the time you would have spent looking for posho [food]. (FGD)

Another health system weakness pointed out as a barrier for HIV testing, was the lack of integration between HIV care and other health services. By singling out and exposing patients seeking HIV care through special clinics or opening hours, men were further discouraged from getting tested.

People start noticing your (HIV) status, because of the specific days you go for check up. (FGD)

While HIV care put patients too much in the limelight, at the ANC the problem was the opposite. Men were excluded from the sessions where their wives were examined, and had to wait outside without any information about what was happening to their pregnant wives. A number of men therefore questioned the rationale for their presence during ANC.

Now the problem with the medical staff is, you as a man, they cannot tell you what exactly they are doing inside there (during ANC), instead I can sit on the verandah and they get on with whatever check-ups and you only take your spouse back home. (IDI)

We are not allowed to go in, it is only the Doctor and the woman, so we are left outside, helpless until the woman comes out/.../. (FGD)

Lack of drugs at the health facilities and general low confidence in the care provided further eroded men's feeling that it was worth their time to go there:

/.../most of the times when you go to the hospital they tell you that there are no drugs they tell you to go to such and such a clinic and buy the drugs/.../. (FGD)

The organizational issues the men cited at the health facilities were compounded by their experience that, in the end, payment was required to really get any medicine. Although most men knew that antiretroviral treatment (ART) was provided for free by several organizations, many feared not having access to care if they tested HIV positive. Even if the drugs were free, some noted, there were additional costs such as transport to treatment sites, leading them to argue that in reality, HIV treatment is only for the rich.

When you join TASO (The AIDS Support Organization, which provides ART for free), you may even fail to get transport to go for medication. So such issues discourage people from going for HIV testing. (FGD)

Even when men did accompany their wives to ANC or attend the health facilities, most men had experienced the demanding of informal payments from health care workers.

The doctors normally demand money for "Sumbusa" literally meaning paying them for their services (all the men in the group laugh since they recognize the scenario). (FGD)

Above and beyond waiting times, lack of drugs, costs, and the demanding for money, however, perhaps the most unpleasant aspect of accompanying their wives to ANC that men discussed was enduring the rude treatment their wives often received at the hands of some health care workers.

Sometimes nurses abuse our wives in our presence saying, "whoever owns this one might be having problems." If a nurse abuses your wife in your presence, you can't go back to that place again. In fact going to the hospital for ANC is not easy but we just endure. In fact most times we are just forced to go the health units but it is not easy. (FGD)

The health workers' mistreatment of the spouses made the men feels uncomfortable and embarrassed. The men's feeling of embarrassment actually seemed to be more important than the fact that women were abused.

You may go with your wife but on reaching there and looking at the way they are treating your wife is unkind, it is not good for the man to be there. These nurses, there is a way these women are treated/.../she is abused and embarrassed. So for us men, we just choose not to accompany our wives to avoid such things. (FGD)

Some men had experienced nurses at ANC speaking disrespectfully also to them directly:

/.../sometimes when you get there (to the ANC clinic) the nurse wants to talk to you in a dominating way just like she talks to the pregnant women. So we sometimes feel like not going there because the nurse will be rude to us yet it is not we men who are pregnant. They use harsh language, the same kind they use with women in labor, so sometimes they want to use that same harsh language with us. Yet we men are easily provoked and could even become harsher than the nurses, so you rather stay away (many participants laugh and give signals of approval). (FGD)

Given all of the above, it is not surprising that seeking health care in general seemed to be rare among these men.

/.../but they will get a few patients because there are people who have take even 40 years without going to the hospital so it's so hard because if someone buys Panadol (pain killers) and feels better... (FGD)

Respondents' reasons for not accepting couple HIV testing, and factors that would make them accept couple testing, are summarized in Table 1.

**Table 1:** The men's reasons for not accepting couple HIV testing, and suggestions for how to increase men's acceptance of couple HIV testing

A. The men's reasons for not accepting couple HIV testing
• Worries and fear related to relationship problems
• Lack of fully understanding of why they should be tested
• Distance to health facilities and waiting time
• HIV is treated as a special disease at health facilities and this is stigmatizing
• Health facilities are not male friendly
• Health workers treat the men and their wives badly
B. Suggestions for how to increase men's acceptance of couple HIV testing

• Community based information campaigns
• Peer influence and role models
• Avoid treating HIV as a special disease at health facilities
• Make HIV testing easier through:
a. More flexible opening hours for testing at health facilities
b. Provide testing from non-governmental organizations
c. Provision of transport to testing site
d. Provision of home-based testing

## Discussion

According to our study, couple HIV testing during ANC is unpopular among rural Ugandan men primarily because they fear relationship conflicts in case of HIV sero-discordance. The men lack incentives to be tested, partner support, or secondary prevention of HIV is not considered as important reasons for testing. Furthermore, the men feel unwelcomed and disrespected at antenatal care facilities.

Set against the background of the actual marriage relationships described by men in our study, the widely implemented policy of couple HIV-testing appears to be based on an unrealistic view of the nature of marriage in rural Uganda. The "promise" of couple HIV testing seemed to make the men less likely, not more likely, to test. The risk of one partner turning up infected and the other not, could easily create suspicion that one or the other partner had been unfaithful, and was thought to threaten marriage stability and family harmony. Previous research in Uganda has shown that marriages can also be easily destabilized by fertility issues such as lack of a male child [27].

On a theoretical level, our study supports previous research that has pointed out how traditional gender norms in themselves can constitute significant barriers to health care seeking among men across the world[28]. The norm that men should not show weakness, for example by seeking health care, dictates against men being HIV tested, especially alongside their wives [28]. Social structures, cultural norms, and implicitly gender-biased health systems stand in complex relationships to one another, and influence health outcomes for men and women differently [29]. Former research has shown that HIV-infected men find it more difficult than HIV-infected women do to disclose their infection directly to their spouse, and that men prefer to use a third party as a mediator [30]. The results from our study provide a robust example of gender as a social determinant of health since men are expected to enter the female domain of the ANC clinic to be tested and counseled alongside their wives. The couple HIV-testing policy runs up against the more common pattern in Uganda of a clear division between male and female domains [25].

Another way in which the interaction of gender and health systems plays out in our research is in the rhetoric of couple HIV testing. The ANC is perceived as largely catering to women, failing to take men's needs into account. The couple HIV testing policy mimics the discourse common when discussing male involvement in sexual and reproductive health programs in general, where men's involvement is introduced to enable women to access health care services, rather than for the men's own good [31-35].

According to men in our study, and also shown in other research, separating HIV testing, care and treatment from other kinds of health care only increases the stigma of living with HIV [36]. Integrating HIV care into general health care would not only make testing, monitoring and ART provision more sustainable, but would also reduce the stigma that research has already related to the vertical organization of HIV care [37]. Alternative HIV testing methods such as mobile clinics, work-place testing opportunities, and door-to-door testing, suggested by our study participants, have also been shown to increase uptake of services and reduce stigma [38,39]. A further factor in men's reluctance to go for testing was the perceived rude behavior among health workers', a factor also found in studies in Tanzania [40,41]. Therefore interventions targeting health workers also seem to be crucial.

The WHO recommends that the implementation of policies encouraging male involvement during ANC should always be accompanied by awareness campaigns in the community, to help men - and women- to understand why the policies are put in place[42]. The policy for couple HIV testing might have created awareness about couple testing among these Ugandan men, but the policy did not make them act and get tested. The men pointed out, for example, that the mass media were their main source of information about HIV, but that they would prefer live sensitization programs in their communities. Several studies in Uganda, and elsewhere in SSA, have found that both sexual health awareness as well as increased testing, more use of condoms, more use of family planning, and less sexual risk-taking, all can be successfully achieved by "live" community, and peer-to-peer interventions [43-45]. One study found that exposure to radio programs about family planning in Uganda did not increase actual contraception except for individuals who also received "live" interpersonal communication[44]. Thus, one-way communication through media can create awareness, but live, personal communication may be needed to deepen men's understanding of the benefits associated with HIV testing and make them act.

The men in our study had several suggestions for how to improve the current couple HIV testing policy: peer sensitization of men, make health facilities less stigmatizing and more male-friendly, train health workers to meet men's needs, and hold discussions between health workers and community members.

A potential weakness of our study was that a female carried out the interviews and some of the FGDs. However, when comparing FGD responses, no differences attributable to the moderator's gender were found. Instead, the men talked very freely and often asked sensitive questions about safe sex, discordance and condom use. Since many answers were neither expected nor socially desirable, we believe that this study offers a fair description of actual attitudes and practices in this population.

## Conclusion

Pursuing couple HIV testing as a main avenue for getting men to be tested and supportive of PMTCT for their wives, seems not to work in its current form. In order to implement a successful, culturally adapted couple testing policy in this setting, our research suggests that gender differences in health-seeking behavior, health systems' different effects on men and women, and gender structures in society at large must be taken into account.

## Competing interests

The authors declare that they have no competing interests.

## Authors' contributions

ECL coordinated the study. ECL, AT, XN and AME designed, planned the data study and developed the data collection tools. ECL, SN, and XN collected the data. All authors took part in data analysis. ECL and XN drafted the first manuscript. All authors read and approved the final manuscript.

## Pre-publication history

The pre-publication history for this paper can be accessed here:

http://www.biomedcentral.com/1471-2458/10/769/prepub
